# Supplementary material for: Evaluation of EV Storage Buffer for Efficient Preservation of Engineered Extracellular Vesicles
Source: Int J Mol Sci. 2023 Aug 16;24(16):12841. doi: 10.3390/ijms241612841 (PMC10454675; doi:10.3390/ijms241612841)
Supplement: Supplementary file 1 [file ijms-24-12841-s001.zip › ijms-2543218-supplementary.pdf]

Supplemental Figures

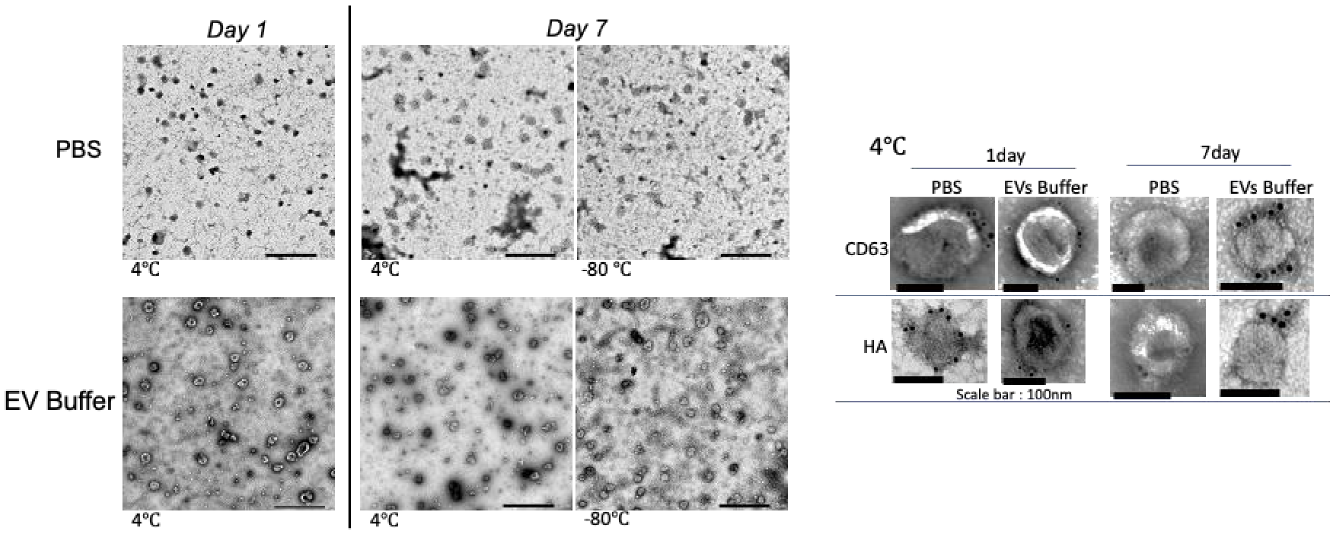

Figure S1. Transmission electron microscopy images of eEVs after the storages in PBS and the EV buffer showing gold-labeled HA and CD63 surface markers.

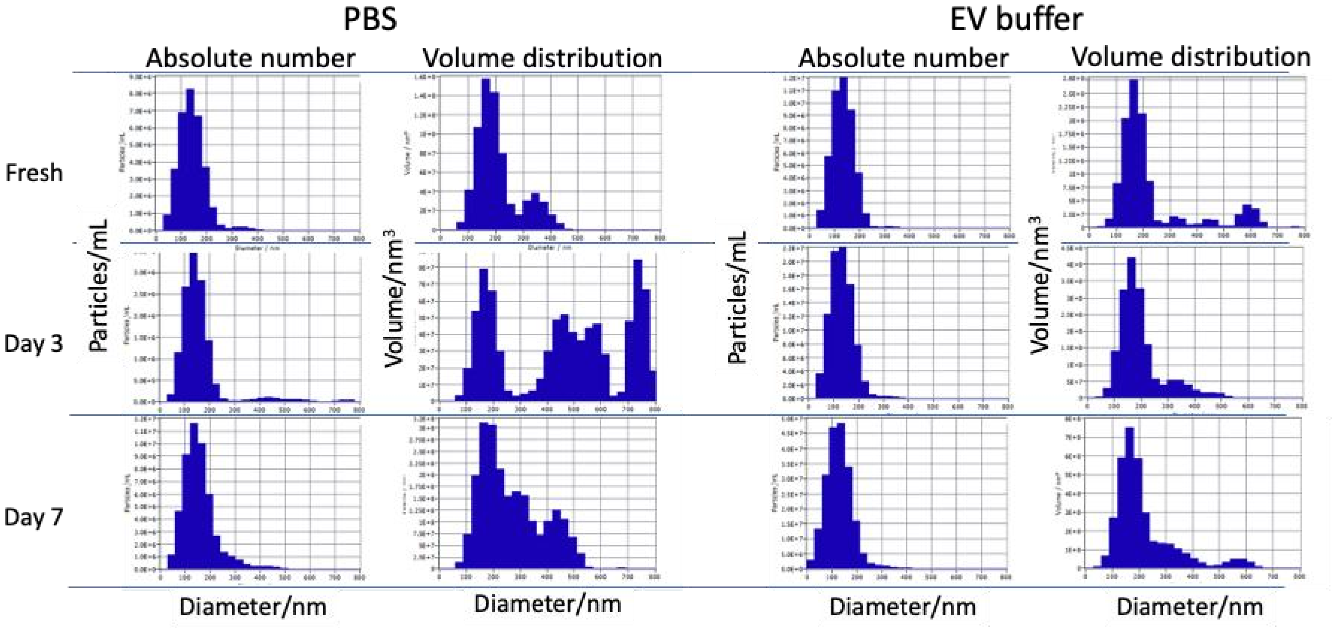

Figure S2. Typical examples of size distribution by different storage conditions. No significant change was observed on absolute number of EVs, PBS showed volume increase of large size particles than EV storage buffer.

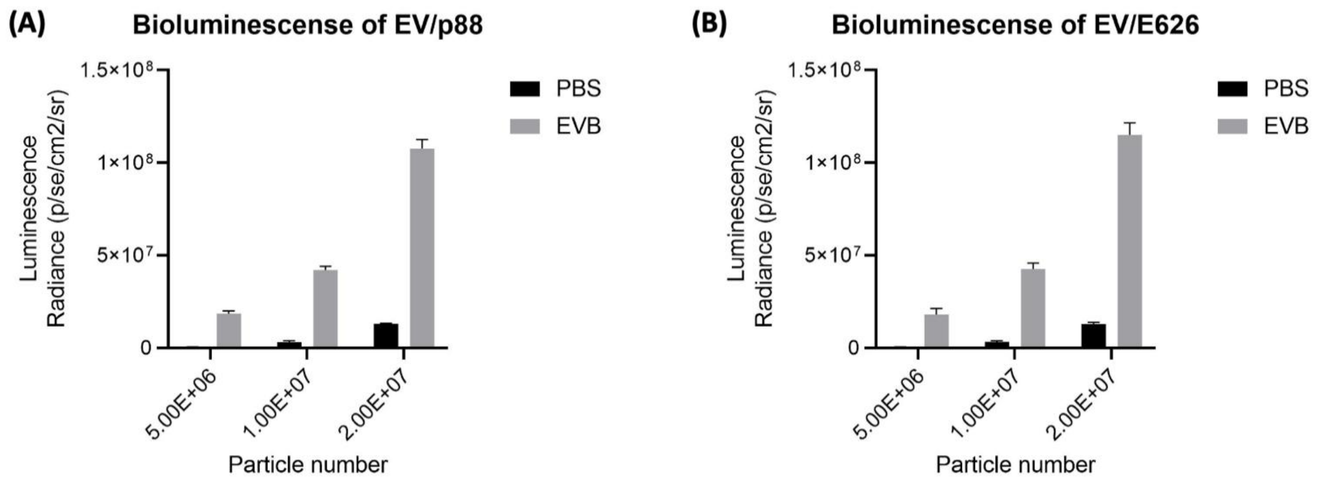

Figure S3. Comparison of eEV bioluminescence in PBS or EV buffer. Bioluminescence of $5x10^6$, 10^7, and $2x10^7$ eEVs co-labeled with gLuc and p88 peptide **(A)** or E626 monobody **(B)** either in PBS or EV buffer were measured following the substrate addition. The total photon flux (p/s) from EVs bound to the cells by IVIS. The value represents the means ± SD (n = 3) in the graph.
